# Changes in Food Insecurity Among Individuals Using a Telehealth and Nutrition Platform: Longitudinal Study

**DOI:** 10.2196/41418

**Published:** 2022-10-25

**Authors:** Shivani Bakre, Benjamin Shea, Kaylee Ortega, Jared Scharen, Jason Langheier, Emily Hu

**Affiliations:** 1 Foodsmart San Francisco, CA United States

**Keywords:** food insecurity, tele-nutrition, telehealth, meal planning, SNAP, diet, nutrition, COVID-19

## Abstract

**Background:**

Food insecurity is a complex public health problem affecting many individuals in the United States. Digital health interventions that promote behavior change and provide access to affordable and healthy food may help to alleviate food insecurity.

**Objective:**

The aim of this study was to characterize food-insecure users of Foodsmart, a telehealth and nutrition platform with meal planning, food ordering, nutrition education, budgeting, and grocery discount features, and to evaluate changes in diet and food insecurity.

**Methods:**

We retrospectively analyzed data collected from 4595 adults who used the Foodsmart platform between February and October 2021. Participants self-reported their diet, demographics, biometrics, and food insecurity status in a 56-item questionnaire. Participants were reported to be food insecure if they answered “sometimes” or “often” to the question “How often does the food you buy not last and you don't have money to get more?” from the United States Department of Agriculture’s Household Food Security survey. We examined baseline characteristics of participants by food insecurity status, associations between characteristics and baseline food insecurity, and changes in diet quality and food insecurity status. To evaluate potential causes of reversing food insecurity, the use of 6 Foodsmart features was compared between food-insecure participants who achieved food security versus food-insecure participants who remained food insecure, based on their last response to the food insecurity question.

**Results:**

We found that 16% (742/4595) of participants were food insecure at baseline. Participants who were food insecure at baseline were more likely to be obese, to have at least one chronic condition, to have a lower diet quality, to cook less frequently at home, to think healthy food is too expensive, and less likely to order takeout or eat at a restaurant. Among participants who were food insecure at baseline, 61% (451/742) improved their nutrition and 29% (217/742) responded that they were food secure at follow-up, with an increasing percentage achieving food security with longer enrollment time. Using a multivariable logistic regression model, we found that age, diabetes, prediabetes, BMI categories, and diet quality at baseline were statistically significantly associated with the likelihood of being food insecure at baseline. Among those who were food insecure at baseline, there was a higher relative proportion of participants who achieved food security and used the “deals” (28.6% higher), “CookItNow” (36.4% higher), and “telenutrition” (27.5% higher) features compared to those who remained food insecure.

**Conclusions:**

This study assesses the characteristics of individuals enrolled on the Foodsmart platform who answered the food insecurity question. We found that a significant number of participants who were food insecure at enrollment achieved food security. This finding shows that telehealth and nutrition platforms may potentially help users improve household food security.

## Introduction

Food insecurity affects many households in the United States, profoundly impacting the health and financial stability of individuals. Recent trends in the last decade have suggested a lower prevalence of food insecurity, dropping from 13% of Americans reported to be food insecure in 2016 to 10.5% in 2019 [1,2]. However, the COVID-19 pandemic has dramatically exacerbated the problem, increasing household food insecurity to 38% in March 2020 [3]. The increase in food insecurity can be attributed to a variety of factors, such as poverty; unemployment; instability and disruptions of the food supply to grocery stores and charitable feeding systems, such as food banks; and lack of eligibility, access, or enrollment in federal programs like the Supplemental Nutrition Assistance Program (SNAP) [4,5]. In a cross-sectional observational study conducted at the end of April 2020, 15.6% of households that were food secure prior to the pandemic experienced low food security during the pandemic, but only 2.3% of households that were food insecure before the pandemic became food secure [6]. A national survey conducted at the end of June 2020 also found that 59% of households in which one member lost a job or income were food insecure [7]. Low food security could also be associated with worsening diet quality and purchasing of unhealthy foods. Adams et al [6] found in a cross-sectional study that about one-third of households that experienced low food security during the pandemic reported increasing the purchasing of high-calorie snack foods, desserts, and sweets. Furthermore, in a cross-sectional analysis of National Health and Nutrition Examination Survey results from 2011 to 2014, food-insecure adults reported a 2.22-unit lower Healthy Eating Index (HEI)-2015 score compared to food-secure adults [8].

The impact of food insecurity on health care expenditures is significant. A model evaluating the impact on health care costs that used 2011 to 2013 National Health Interview Survey/Medical Expenditure Panel Survey data estimated that food-insecure adults spent US $1834 more on health care annually compared to food-secure adults; this equates to an estimated US $51.8 billion in excess health care expenditures due to food insecurity in 2016 [1].

A major contributor to exorbitant health care expenditures is the high prevalence of chronic conditions among individuals with food insecurity. A United States Department of Agriculture (USDA) report found that among adults with very low food security, there were 16.4%, 7.2%, 3.6%, and 3.7% higher proportions of hypertension, diabetes, coronary heart disease, and kidney disease, respectively, compared to adults with high food security [9]. Obesity and higher BMI have also been found to be associated with food insecurity [10]. Additionally, a study conducted on 711 patients with diabetes by the American Diabetes Association showed that food-insecure participants were more likely to have poor glycemic control and were more likely to report difficulties affording a diabetes-friendly diet than those who were food secure [11].

There are significant opportunities for interventions to alleviate food insecurity through increasing food access. Prior studies have evaluated the impact of food delivery and online grocery shopping on food insecurity and diet. The Community Servings: Food as Medicine for Diabetes clinical trial, where participants were delivered medically tailored meals in a randomized crossover study, reported that 42% of participants were food insecure during the on-meal-delivery periods versus 62% during the off-meal-delivery periods [12]. The Baltimore Virtual Supermarket Program, a large-scale online grocery ordering system in which participants can pick up their groceries from a local hub, showed that among 93 survey respondents, 93% believed the program made it easier for them to eat healthily and 61% attributed access to healthy foods to the program [13].

Digital technology can serve as an intervention to help alleviate food insecurity. Foodsmart, a telehealth provider platform with a large network of registered dietitians (RDs) across the country that includes a digital nutrition platform, is a potential solution to help address food insecurity, diet quality, and health outcomes. The platform provides personalized recipe recommendations and meal planning and helps users purchase ingredients and compare prices between participating grocers. The platform also allows users to use SNAP benefits to purchase foods online. Previous research on Foodsmart found improvements in clinical metrics, such as weight, lipid levels, and hemoglobin A_1c_, among users with obesity, dyslipidemia, and diabetes, respectively, suggesting clinical benefits of the platform [14-17].

The objective of this study was to characterize demographics, meal planning characteristics, and diet quality among participants who were food insecure compared to participants who were food secure. The study also sought to evaluate longitudinal associations between using Foodsmart and changes in diet quality and food insecurity status among participants during the COVID-19 pandemic.

## Methods

### Study Sample

**Figure 1:**
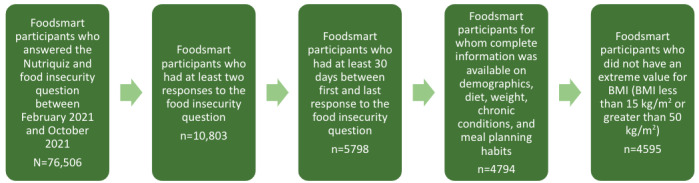
Participant selection criteria.

As of October 2021, 76,506 users of Foodsmart across the United States had answered a question on their food insecurity status. Participants were connected and enrolled into the Foodsmart platform as a service, either through their employer or insurance. We included in our analysis participants who had answered this question at least 2 times since its implementation in February 2021, with their last response being at least 30 days after their first response (n=5798) and for whom complete information was available on demographics, diet, weight, chronic conditions, and meal planning habits (n=4794). Participants who had extreme values for BMI (<15 kg/m^2^ or >50 kg/m^2^) were excluded from our sample (n=199). Our final study sample was 4595 participants (Figure 1).

### Foodsmart, a Telehealth and Nutrition Platform

Foodsmart is a platform that includes two components: (1) a digital app and web solution with tools including nutrition assessments, meal planning, and grocery ordering and (2) a telehealth component that lets users virtually meet with an RD. These components are incorporated into 6 features of the Foodsmart platform: “CookItNow,” “recipes,” “meal planner,” “food buying,” “deals,” and “telenutrition.” The CookItNow feature prompts the user to input ingredients they currently have in their kitchen; the Foodsmart platform then provides them tailored recipes based on those ingredients. The recipes feature on the Foodsmart app also allows individuals to filter recipes based on their food preferences or by category, such as budget-friendly recipes, recipes with leafy greens, or recipes fit for a SNAP budget. The meal-plan feature allows users to create a personalized meal plan based on the user’s dietary assessment, built from thousands of recipes. The food-buying feature then allows the user to have their meal plan automatically transferred to a grocer for online grocery pickup or delivery. The Food and Nutrition Service SNAP Online Purchasing Pilot [18] has also enabled individuals to pay for groceries with SNAP benefits, encouraging individuals to spend SNAP dollars on nutritious food. Foodsmart also offers medically tailored meals on its platform that users can order or that health plans can subsidize. The deals feature helps individuals save approximately 34% on each grocery order via (1) providing digital coupons from the grocery store they shop at and (2) finding the store with the cheapest price for their groceries. Finally, the telenutrition feature allows participants to meet with RDs who can help them by providing recommendations and support based on their eating and health goals and by providing technology assistance. Foodsmart also includes a food insecurity screening question to help identify users who are food insecure; this helps organizations determine which users need the most help and measure these users’ food insecurity status over time.

### Assessment of Diet and Other Characteristics

Participants report their age, gender, usual dietary intake, meal planning habits, weight, chronic conditions, and physical activity on Foodsmart’s platform by filling out a 56-item modified food frequency questionnaire called Nutriquiz (adapted from the National Cancer Institute Diet History Questionnaire I) upon registration [19]. In addition to dietary questions, the Nutriquiz also ascertains demographic information, height, weight, chronic conditions (including diabetes, prediabetes, high blood pressure, dyslipidemia, or the absence of these conditions), frequency of physical activity (light, moderate, or vigorous), and meal planning habits (frequency of home-cooked meals, frequency of purchasing groceries from a grocery store, and how groceries are bought). We collapsed and renamed the categories of responses for each question to “rarely,” “weekly,” and “daily.” The “rarely” category contains the survey responses “never,” “1 time/month,” and “2-3 times/month.” The weekly category contains the survey responses “1 time/week,” “2 times/week,” “3-4 times/week,” and “5-6 times/week.” The daily category contains survey responses for “1 time/day,” “2-3 times/day,” “4-5 times/day,” and “6+ times/day.”

Based on a participant’s responses to the Nutriquiz questions assessing diet, a score called the Nutriscore was calculated to assess overall diet quality; this score is based on the Alternative HEI-2010 and the Commonwealth Scientific and Industrial Research Organization Healthy Diet Score [20,21]. The Nutriscore has 7 components (called Nutriscore Essentials): fruits, vegetables, protein ratio, fat ratio, carbohydrate ratio, hydration, and sodium. Each component is scored on a scale of 0 to 10, and the scores are then added together for an overall Nutriscore (ranging from 0 to 70). Higher scores indicate higher diet quality. Change in Nutriscore was calculated by taking the difference between a participant’s last Nutriscore and first Nutriscore, given that they were taken at least 30 days apart. An increase in Nutriscore demonstrates that the participant has improved their diet quality.

### Ascertainment of Food Insecurity Status

**Figure 2:**
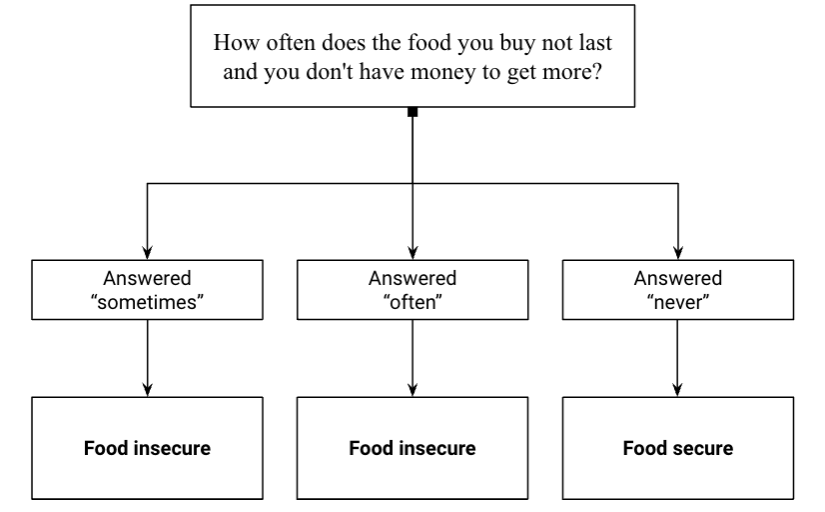
Decision tree for assessing food insecurity status.

In the Nutriquiz, participants answered a question about their level of food insecurity. This question is adapted from one of 2 questions in a shortened food security screener validated by Hager et al [22] that is valid when compared to the USDA 18-item Household Food Security Survey (HFSS) [23]. The question asked was “How often does the food you buy not last and you don’t have money to get more?” The answer choices were “sometimes,” “often,” and “never.” If participants answered “sometimes” or “often” they would be considered food insecure; if they answered “never” to the question, they would be considered food secure, as shown in Figure 2.

### Statistical Analysis

We used descriptive statistics to evaluate participant demographic and clinical characteristics, diet quality, physical activity, and meal planning habits. Categorical variables are presented as percentages of the study population and continuous variables are presented as the mean (SD). We used the chi-square test to determine if there was a statistically significant difference in categorical variables with food insecurity status. We used a 2-sample, 2-tailed *t* test to determine if there was a statistically significant difference in continuous variables with food insecurity status.

We used a multivariable logistic regression model to estimate the odds ratios (ORs) and 95% CIs of baseline food insecurity status for several variables, including gender, age, diabetes, prediabetes, high blood pressure, dyslipidemia, good health (ie, no conditions), baseline BMI category, and baseline Nutriscore. For those who were categorized as food insecure, we calculated the percentage of participants whose status changed to food secure in total and by cumulative length of enrollment in Foodsmart (with cutoffs at ≥2, ≥4, and ≥6 months). To evaluate possible causes for reduction in food insecurity among food-insecure participants at baseline, we compared the difference in the percentage of participants who used 6 Foodsmart features (CookItNow, deals, telenutrition, meal planner, recipes, and food buying) among those who achieved food security versus those who remained food insecure. The absolute difference was calculated by taking the proportion of participants who were food insecure at baseline and became food secure and subtracting the proportion of participants who were food insecure at baseline and remained food insecure. The relative difference was calculated by dividing the absolute difference by the proportion of participants who were food insecure at baseline and remained food insecure.

*P* values of .05 or less were considered to be statistically significant. Stata (version 16; StataCorp) was used for all statistical analyses.

### Ethical Considerations

The study was declared exempt from institutional review board oversight by the Pearl Institutional Review Board given the retrospective design of the study and the less than minimal risk to participants (Protocol #20-ZIPO-101).

## Results

### Participant Characteristics

In order to better understand baseline demographic and clinical characteristics, as well as dietary habits and behaviors, we conducted descriptive analyses stratified by baseline food insecurity (Table 1).

**Table 1:** Baseline characteristics by baseline food insecurity status. The chi-square test was used to test differences in categorical variables.

Characteristics				Total (N=4595), n (%)		Food insecure (N=742), n (%)		Food secure (N=3853), n (%)		*P* value	
Female				3558 (77.4)		609 (82.1)		2949 (76.5)		.001	
**Age**											<.001
	<40 years old			1499 (32.6)		285 (38.4)		1214 (31.5)			
	40-59 years old			2417 (52.6)		380 (51.2)		2037 (52.9)			
	≥60 years old			679 (14.8)		77 (10.4)		602 (15.6)			
**BMI category**											<.001
	Normal (<25 kg/m^2^)			1459 (31.8)		149 (20.1)		1310 (34)			
	Overweight (25-29.9 kg/m^2^)			1393 (30.3)		213 (28.7)		1180 (30.6)			
	Obese (≥30 kg/m^2^)			1743 (37.9)		380 (51.2)		1363 (35.4)			
**Chronic conditions**											
	Diabetes			250 (5.4)		61 (8.2)		189 (4.9)		<.001	
	Prediabetes			239 (5.2)		63 (8.5)		176 (4.6)		<.001	
	High blood pressure			765 (16.6)		159 (21.4)		606 (15.7)		<.001	
	Dyslipidemia			888 (19.3)		156 (21)		732 (19)		.2	
	Healthy (defined as having no conditions)			3048 (66.3)		446 (60.1)		2602 (67.5)		<.001	
**Physical activity**											
	**Light**										<.001
		Almost never		602 (13.1)	130 (17.5)		472 (12.3)				
		Weekly		2379 (51.8)	373 (50.3)		2006 (52.1)				
		Daily		1614 (35.1)	239 (32.2)		1375 (35.7)				
	**Moderate**										<.001
		Almost never		877 (19.1)	192 (25.9)		685 (17.8)				
		Weekly		2820 (61.4)	434 (58.5)		2386 (61.9)				
		Daily		898 (19.5)	116 (15.6)		782 (20.3)				
	**Vigorous**										.01
		Almost never		1537 (33.4)	285 (38.4)		1252 (32.5)				
		Weekly		2621 (57)	395 (53.2)		2226 (57.8)				
		Daily		437 (9.5)	62 (8.4)		375 (9.7)				
**Alcohol use**											<.001
	Almost never			2619 (57)		483 (65.1)		2136 (55.4)			
	Weekly			1663 (36.2)		206 (27.8)		1457 (37.8)			
	Daily			313 (6.8)		53 (7.1)		260 (6.7)			
**Responses to questions on meal planning habits**											
	**In the last 6 months, how often were meals home-cooked by you or a household member?**										<.001
			Almost never	98 (2.1)	28 (3.8)		70 (1.8)				
			Weekly	2216 (48.2)	405 (54.6)		1811 (47)				
			Daily	2281 (49.6)	309 (41.6)		1972 (51.2)				
	**In the last 6 months, how often did you or your household purchase groceries from a grocery store?**										.001
			Almost never	635 (13.8)	134 (18.1)		501 (13)				
			Weekly	3960 (86.2)	608 (81.9)		3352 (87)				
	**How do you typically buy groceries?**										.1
			At a store	3655 (79.5)	568 (76.5)		3087 (80.1)				
			Online	690 (15)	123 (16.6)		567 (14.7)				
			Both	250 (5.4)	51 (6.9)		199 (5.2)				
			With an Electronic Benefits Transfer card	161 (3.5)	65 (8.7)		96 (2.5)		<.001		
	**How often do you feel that healthy food is too expensive to buy?**										<.001
			Never	1883 (41)	48 (6.5)		1835 (47.6)				
			Sometimes	1978 (43)	321 (43.3)		1657 (43)				
			Often	734 (16)	373 (50.3)		361 (9.4)				
	**How often do you order take out/visit a restaurant/fast food establishment?**										.001
			Less than once a week	1917 (41.7)	352 (47.4)		1565 (40.6)				
			At least once a week	2678 (58.3)	390 (52.6)		2288 (59.4)				

We found that 16.2% (742/4595) of participants were categorized as food insecure at baseline. In our sample of 4595 participants, participants who were food insecure at baseline were more likely to be female, be aged 40 to 59 years, be obese, have diabetes, have prediabetes, and have high blood pressure compared to those who were food secure. Additionally, food-insecure participants were less likely to exercise or to drink alcohol. In regard to meal planning habits, participants who were food insecure were less likely to make home-cooked meals on a daily basis, less likely to purchase groceries on a weekly basis, more likely to think that healthy food is too expensive, and less likely to order takeout food or eat at a restaurant or fast-food establishment compared to those who were food secure at baseline. Lastly, a higher percentage of food-insecure participants reported having an Electronic Benefits Transfer card compared to food-secure participants at baseline.

### Changes in Diet Quality by Food Security Status

To better understand how baseline diet quality and change in diet quality were associated with baseline food insecurity status, we show the first and last Nutriscores in Table 2. First and last Nutriscores were significantly higher for those who were food secure compared to food insecure (*P*<.001). However, there was no significant difference in the change in Nutriscore between those who were food insecure and food secure (1.9 points vs 1.4 points).

We show a comparison of the average change in Nutriscore and the subcomponents of the Nutriscore in Table 3. We found that on average, participants who were food insecure at baseline had a greater improvement in overall Nutriscore, vegetable intake, fruit intake, carbohydrate ratio, fat ratio, and protein ratio.

**Table 2:** Baseline and changes in diet quality by food insecurity status. Numbers in parentheses in row headings indicate score ranges; higher numbers indicate higher quality.

Nutriscore		Total (N=4595)	Food insecure (N=742)	Food secure (N=3853)	*P* value
Baseline Nutriscore (0-70), mean (SD)		33.9 (8.6)	31.9 (8.3)	34.3 (8.6)	<.001
**Baseline Nutriscore component scores (0-10), mean (SD)**					
	Vegetables	3.4 (1.4)	3.3 (1.5)	3.5 (1.4)	.002
	Fruits	3.1 (2.2)	2.9 (2.2)	3.2 (2.2)	.003
	Carbohydrate ratio^a^	7.7 (1.7)	7.3 (1.7)	7.7 (1.7)	<.001
	Fat ratio^b^	3.0 (2.8)	2.6 (2.6)	3.1 (2.9)	<.001
	Protein ratio^c^	7.0 (3.2)	6.8 (3.2)	7.1 (3.2)	.03
	Sodium	7.1 (4.0)	6.6 (4.3)	7.2 (3.9)	<.001
	Hydration	6.5 (2.3)	6.3 (2.4)	6.6 (2.2)	.01
Final Nutriscore (0-70), mean (SD)		35.4 (8.5)	33.8 (8.6)	35.7 (8.5)	<.001
Change in Nutriscore, mean (SD)		1.5 (6.9)	1.9 (7.2)	1.4 (6.9)	.1
Participants who improved nutrition at all, n (%)		2690 (58.5)	451 (60.8)	2239 (58.1)	.2
Participants who improved nutrition by 5% or more, n (%)		2173 (47.3)	374 (50.4)	1799 (46.7)	.1

^a^Carbohydrate ratio: total carbohydrate intake divided by total fiber intake.

^b^Fat ratio: polyunsaturated fatty acid intake divided by the sum of intake of saturated and trans fats.

^c^Protein ratio: white meat and plant protein intake divided by red meat and processed meat intake.

**Table 3:** Mean group percentage change in Nutriscore and Nutriscore component scores by food insecurity status at baseline.

Scores		Change in score in food-secure group, %	Change in score in food-insecure group, %
Nutriscore		4	6
**Nutriscore component scores**			
	Vegetables	3.2	3.7
	Fruits	5.1	7.9
	Carbohydrate ratio^a^	1.4	1.6
	Fat ratio^b^	7.2	11.8
	Protein ratio^c^	1	2.2
	Sodium	0.6	–1.7
	Hydration	2.7	0.4

^a^Carbohydrate ratio: total carbohydrate intake divided by total fiber intake.

^b^Fat ratio: polyunsaturated fatty acid intake divided by the sum of intake of saturated and trans fats.

^c^Protein ratio: white meat and plant protein intake divided by red meat and processed meat intake.

### Changes in Food Insecurity Status

To evaluate how food insecurity status changed over time, we determined the proportion of participants who were categorized as food insecure at baseline and were also food secure at the time of their last response among the total population and by cumulative length of enrollment (≥2, ≥4, and ≥6 months). Of the 742 food-insecure participants at baseline, 29.2% (217/742) were food secure at the time of their last response. When subsetting participants by cumulative length of enrollment (≥2 months, ≥4 months, and ≥6 months), 30.9% (182/590), 33% (70/212), and 42.4% (25/59) of participants, respectively, changed from food insecure to secure. We also evaluated the proportion of participants who were categorized as food secure at baseline and were subsequently food insecure in their last response. Of the 3853 food-secure participants at baseline, 6.7% (259) were food insecure at the time of their last response.

### Characteristics Associated With Food Insecurity Status at Baseline

In order to evaluate which participant characteristics were associated with food insecurity at baseline, we used a multivariable logistic regression model adjusted for demographics, chronic conditions, and baseline diet quality (Table 4). We found that females were 44% more likely to be food insecure compared to males (OR 1.44, 95% CI 1.17-1.77; *P*=.001). Compared to those who were younger than 40 years, those aged 40 to 59 years were 34% less likely to be food insecure (OR 0.66, 95% CI 0.55-0.79; *P*<.001), and those aged at least 60 years were 56% less likely to be food insecure (OR 0.44, 95% CI 0.33-0.59; *P*<.001). Participants with diabetes were 65% more likely to be food insecure compared to those without diabetes (OR 1.65, 95% CI 1.17-2.32; *P*=.004). Participants with prediabetes were 61% more likely to be food insecure compared to those without prediabetes (OR 1.61, 95% CI 1.14-2.27; *P*=.01). Additionally, those who were overweight or obese were, respectively, 60% (OR 1.60, 95% CI 1.28-2.02; *P*<.001) and 118% (OR 2.18, 95% CI 1.75-2.71; *P*<.001) more likely to be food insecure than those who had a normal BMI. Finally, for each 5-point increase in a participant’s baseline Nutriscore, they were 10% less likely to be food insecure (OR 0.90, 95% CI 0.86-0.95; *P*<.001).

**Table 4:** Multivariable logistic regression assessing factors associated with baseline food insecurity status.

Characteristics		Odds ratios (95% CI)	*P* value
Gender (female)		1.44 (1.17-1.77)	.001
**Age**			
	<40 years	1 (reference)	
	40-59 years	0.66 (0.55-0.79)	<.001
	≥60 years	0.44 (0.33-0.59)	<.001
**Conditions**			
	Diabetes	1.65 (1.17-2.32)	.004
	Prediabetes	1.61 (1.14-2.27)	.01
	High blood pressure	1.25 (0.94-1.67)	.1
	Dyslipidemia	0.99 (0.75-1.32)	.9
	Healthy (no conditions)	1.01 (0.72-1.41)	.9
**BMI category**			
	Normal	1 (reference)	
	Overweight	1.60 (1.28-2.02)	<.001
	Obese	2.18 (1.75-2.71)	<.001
Baseline Nutriscore (per 5 points)		0.90 (0.86-0.95)	<.001

### Foodsmart Platform Usage Among Participants Who Were Food Insecure at Baseline

To evaluate possible causes of overall reduction of food insecurity among food-insecure participants, we compared the percentage of users who used specific Foodsmart features among food-insecure participants who achieved food security and food-insecure participants who remained food insecure (Table 5). There was a 36.4% higher relative proportion of participants who used the CookItNow feature, a 28.6% higher relative proportion of participants who used the deals feature, and a 27.5% higher relative proportion of participants who used the telenutrition feature among participants who achieved food security versus participants who remained food insecure.

**Table 5:** Proportion of participants who were food insecure at baseline who used the Foodsmart feature by last food security status.

Foodsmart features	Users who achieved food security who used the feature (N=217), n (%)	Users who remained food insecure who used the feature (N=525), n (%)	Absolute difference, percentage points	Relative difference, %
CookItNow	48 (22.1)	85 (16.2)	5.9	36.4
Deals	43 (19.8)	81 (15.4)	4.4	28.6
Telenutrition	11 (5.1)	21 (4)	1.1	27.5
Meal planner	64 (29.5)	155 (29.5)	0	0
Recipes	192 (88.5)	477 (90.9)	–2.4	–2.6
Food buying	145 (66.8)	372 (70.9)	–4	–5.8

## Discussion

### Principal Findings

In this study of 4595 participants, 16% (742) were identified as being food insecure at baseline. Participants who were food insecure at baseline were more likely to be female, older, have a preexisting condition (overweight or obesity, diabetes, or prediabetes), have a lower quality diet, and believe healthy food is too expensive. Additionally, those who were food insecure were less likely to exercise, consume alcohol, prepare home-cooked meals daily, order takeout or eat at a restaurant at least once a week, or buy groceries compared to those who were food secure at baseline. Among participants who were food insecure at baseline, 29% (217/742) identified as being food secure at the end of follow-up.

Diet quality, assessed using the Nutriscore, improved in both the food-insecure and food-secure groups by 1.9 and 1.4 points on average, respectively; this suggests that the platform can benefit food-insecure and food-secure participants equally in improving nutrition. Furthermore, both groups improved in most of the components that make up the Nutriscore, with greater improvements in the food-insecure group most likely due to lower scores on average at baseline compared to scores in the food-secure group.

In our multivariable logistic regression evaluating the association between various characteristics and food insecurity at baseline, we found that those who were female, were younger than 40 years old, had diabetes or prediabetes, or were overweight or obese were more likely to have food insecurity. For every 5-unit increase in baseline diet quality as assessed by Nutriscore, the likelihood of a participant being food insecure was 10% lower. This aligns with a study conducted by Leung et al [24], which found that food insecurity was associated with lower diet quality. Furthermore, these results are consistent with another study assessing trends in food insecurity in the United States, which found that individuals aged 65 years or older were less likely to be food insecure than individuals who were 18 to 34 years old, and females were 23% more likely to be food insecure than males [25].

In evaluating which Foodsmart features participants used, we found that a higher proportion of participants who were food insecure at baseline and achieved food security used the CookItNow, deals, and telenutrition features than participants who were food insecure at baseline and remained food insecure. The CookItNow feature promotes cooking at home more often with ingredients participants already have at home, showing that there could be a potential association between cooking at home and food security. The use of the deals feature to find the best prices for grocery ingredients can also help participants spend less on their food purchasing, which could also potentially lead to reducing food insecurity. The use of telenutrition visits with an RD to better understand how to cook and meal plan on a budget while eating healthily could also potentially support reducing food insecurity.

These results align with those of prior studies, which demonstrated a higher prevalence of obesity and fewer home-cooked meals among those who were food insecure [26,27]. Pan et al [26] found that among adults in 12 states in the United States, the prevalence of obesity was significantly higher among food-insecure adults than among food-secure adults (35.1% vs 25.2%; *P*<.001). A hypothesis for explaining this association is that food-insecure individuals are more likely to consume inexpensive, energy-dense foods. Furthermore, a cycle of having an abundance of food at the beginning of the month followed by food scarcity at the end of the month, due to the monthly distribution of SNAP benefits, may contribute to weight gain [28]. In a study of 1171 SNAP-eligible adults in Texas, Ranjit et al [27] found that food-secure participants had more days where they ate a home-cooked meal (difference of –0.26 days, *P*=.03) and cooked more days per week (difference of –0.30 days, *P*=.01) than food-insecure participants. A lower frequency of cooking and eating at home among food-insecure participants compared to food-secure participants may be due to less access to cooking supplies and resources and less time. This highlights the importance of increasing nutrition and cooking knowledge and increasing resources to help promote cooking within a limited budget and time constraints.

In line with our findings, a prior study showed that medically tailored meal delivery for participants with diabetes improved food insecurity and dietary quality [12]. The Food as Medicine for Diabetes clinical trial used a 24-week randomized cross-over design to study 42 adults with type 2 diabetes who reported food insecurity based on a 2-item screener questionnaire [22]. Dietary quality improved (the mean HEI-2010 score was 71.3 during the on-meal-delivery period, compared to 39.9 during the off-meal-delivery period; *P*<.001). Furthermore, participants reported a 20% lower prevalence of food insecurity during on-meal-delivery periods versus off-meal-delivery periods (*P*=.047). While our study was a 1-sample pre-post analysis, our results were in line with this randomized cross-over clinical trial. However, our study did not specifically use medically tailored, specific delivery. With the cost of food per month being on average US $281 per individual in the United States [29], it is also uncertain whether these medically tailored meal plans are cost-effective for a larger population, given that the cost of the meals and delivery was approximately US $350 per individual per month in this study [12].

Addressing food and nutrition insecurity requires solving problems for both food access and affordability. However, affordability and access need to be applied not just to food in general, but specifically to nutritious food, as ultraprocessed foods with poor nutritional quality exacerbate the prevalence of chronic conditions [30]. Affordability has been enhanced by a record SNAP increase in 2021 [31], but many families still struggle with affording food outside of the SNAP allowance. Additionally, many of these funds are often allocated toward nonnutritious foods [32]. Many individuals with food insecurity struggle with disabilities or lack of transportation, limiting their ability to get to a physical grocery store [33]. Many major online grocers have begun accepting SNAP benefits online; however, for many of these users, the online grocery delivery fees are still prohibitive. Additionally, others are unaware if they qualify for SNAP and struggle to navigate the SNAP enrollment process. A prior study in California showed that newly enrolled participants in SNAP were less likely to report food insecurity (83.1% vs 67.5%, *P*<.001), showing that SNAP enrollment can reduce food insecurity [34]. Many health plans have also attempted to deliver meals to solve food insecurity, but unfortunately, this has been difficult to operationalize and scale in a cost-effective manner; for example, the Blue Cross Blue Shield Association terminated its FoodQ pilot after 1 year [35]. In our study, 29% of food-insecure users at baseline reported becoming food secure, and diet quality improved over time; thus, the Foodsmart platform is addressing these food insecurity challenges at scale. Foodsmart RDs have also since begun enrolling members in SNAP, and we plan to evaluate the impact of SNAP enrollment on long-term food insecurity status in a future study.

### Strengths and Limitations

This study had several important strengths. To our knowledge, no longitudinal study has been conducted at this scale to assess the impact of a telehealth platform or nutrition and meal planning app on food insecurity and diet quality. A systematic review of interventions addressing food insecurity in a health care setting found that very few studies that evaluated health outcomes had high enrollment or a significant follow-up period [36]. Our study differs from these studies in that it evaluated postintervention outcomes in dietary intake and food insecurity status and had high enrollment and a long-term follow-up period (mean length 3.3, SD 1.5, months). Given the broad range of follow-up times, we were able to measure changes in food insecurity over differing lengths of time. The question assessing food insecurity was added in February 2021, and almost 5000 users responded to it twice within 8 months, showing the power of digital technology to collect large amounts of data in real time in response to public health crises. To assess food insecurity, we used a question adapted from the USDA’s 18-item HFSS that has been previously validated. Last, we were able to draw associations between food insecurity and meal planning and eating habits, which are important factors in food security and nutrition.

There are some important limitations to note for this study. This study only used 1 of 18 questions on the 18-item HFSS to evaluate food insecurity [23]. Due to the length of time needed to complete the 18-item HFSS, the first question was chosen as an efficient method to assess food insecurity. Hager et al [22] found that 92.5% of respondents from food-insecure households agreed with the statement “Within the past 12 months we worried whether our food would run out before we got money to buy more.” While our study only used the first question, this question was still a strong indicator of food insecurity, and the second question, as used by Hager et al [22], has been recently added. Since we did not have exact data on when participants left the program, we used the last response to the food insecurity question as an approximate end date. Furthermore, it is challenging to draw firm conclusions on how duration of usage was associated with change in food insecurity because we are using real-world data without the consistent setting of a controlled study; participants were free to start and stop usage of the app whenever they wanted to. Due to the observational design of this study, we cannot draw any causal conclusions on whether usage of the platform leads to transitioning to food security.

### Conclusions

This study evaluated changes in self-reported food insecurity status and diet quality among participants with food insecurity who used a telehealth and nutrition platform with personalized recipe recommendations, meal planning, food ordering, grocery discounts, and price comparisons. While associations can be drawn between the use of Foodsmart features and achieving food security and better nutrition, future research, including a randomized controlled trial, will be needed to assess the causal effect of the Foodsmart platform on dietary changes and reduction in food insecurity.

## Abbreviations

HEI: Healthy Eating Index
HFSS: Household Food Security Survey
OR: odds ratio
RD: registered dietitian
SNAP: Supplemental Nutrition Assistance Program
USDA: United States Department of Agriculture
